# Distinct Patterns of DNA Damage Response and Apoptosis Correlate with Jak/Stat and PI3Kinase Response Profiles in Human Acute Myelogenous Leukemia

**DOI:** 10.1371/journal.pone.0012405

**Published:** 2010-08-25

**Authors:** David B. Rosen, Santosh Putta, Todd Covey, Ying-Wen Huang, Garry P. Nolan, Alessandra Cesano, Mark D. Minden, Wendy J. Fantl

**Affiliations:** 1 Princess Margaret Hospital, Toronto, Ontario, Canada; 2 Baxter Laboratory for Stem Cell Biology, Department of Microbiology and Immunology, Stanford University, Stanford, California, United States of America; 3 Nodality, Inc., South San Francisco, California, United States of America; Yale Medical School, United States of America

## Abstract

**Background:**

Single cell network profiling (SCNP) utilizing flow cytometry measures alterations in intracellular signaling responses. Here SCNP was used to characterize Acute Myeloid Leukemia (AML) disease subtypes based on survival, DNA damage response and apoptosis pathways.

**Methodology and Principal Findings:**

Thirty four diagnostic non-M3 AML samples from patients with known clinical outcome were treated with a panel of myeloid growth factors and cytokines, as well as with apoptosis-inducing agents. Analysis of induced Jak/Stat and PI3K pathway responses in blasts from individual patient samples identified subgroups with distinct signaling profiles that were not seen in the absence of a modulator. *In vitro* exposure of patient samples to etoposide, a DNA damaging agent, revealed three distinct “DNA damage response (DDR)/apoptosis” profiles: 1) AML blasts with a defective DDR and failure to undergo apoptosis; 2) AML blasts with proficient DDR and failure to undergo apoptosis; 3) AML blasts with proficiency in both DDR and apoptosis pathways. Notably, AML samples from clinical responders fell within the “DDR/apoptosis” proficient profile and, as well, had low PI3K and Jak/Stat signaling responses. In contrast, samples from clinical non responders had variable signaling profiles often with *in vitro* apoptotic failure and elevated PI3K pathway activity. Individual patient samples often harbored multiple, distinct, leukemia-associated cell populations identifiable by their surface marker expression, functional performance of signaling pathway in the face of cytokine or growth factor stimulation, as well as their response to apoptosis-inducing agents.

**Conclusions and Significance:**

Characterizing and tracking changes in intracellular pathway profiles in cell subpopulations both at baseline and under therapeutic pressure will likely have important clinical applications, potentially informing the selection of beneficial targeted agents, used either alone or in combination with chemotherapy.

## Introduction

Proteomic technologies that can monitor aberrant cell signaling in disease hold promise in enabling more accurate diagnosis and prognosis, as well as predicting response to therapeutic agents [1]–[3]. Single cell network profiling (SCNP) utilizing flow cytometry differs from most proteomic technologies by measuring modulated phospho-protein and other signaling protein responses at the single cell level [3]–[4]. Several studies have shown that in hematological malignancies, induced protein phosphorylation was more informative than its frequently measured basal phosphorylation state, revealing signaling deregulation consequent to the numerous molecular changes characteristic of transformed cells [5]–[8]. Profiling at the single cell level allows deregulated pathways to be identified in rare cell populations which would otherwise be missed by alternative technologies. Acute Myeloid Leukemia (AML) is characterized by uncontrolled proliferation of myeloid progenitors in the bone marrow [9]–[10]. An accretion of genetic alterations in these cells arrests their normal differentiation and results in a clinically heterogeneous disease challenging successful treatment [11]–[15]. The net result of these molecular changes is alteration of proteins within signal transduction networks that drive functional changes in cell proliferation, survival, differentiation, progression and cellular responses to drug therapy [16]–[21].

Supporting this, a prior study classified AML disease via a series of *in vitro* functional performance tests in response to a panel of myeloid growth factors and cytokines that were individually applied to AML samples [5]. In that study, a limited set of AML cellular response profiles were revealed, most notably potentiated p-Stat3/p-Stat5 signaling post stimulation with G-CSF, which was associated with a negative outcome for patients receiving standard AML chemotherapy. These data corroborate many studies describing a strong tie between the Jak/Stat signaling pathway with tumorigenesis, especially in myeloid malignancies such as juvenile myelomonocytic leukemia and myeloproliferative neoplasms [22]–[25]. The involvement of Jak/Stat signaling in tumorigenesis is plausible, since phosphorylation and dimerization of Stat proteins results in their translocation to the nucleus where they activate a variety of transcriptional programs, including gene sets involved in cell cycle progression and survival [22]–[23], [26]–[27].

In the AML study by Irish, although a subset of samples were identified in which potentiated p-Stat3/p-Stat5 signaling correlated with clinical refractoriness to chemotherapy, not all AML patients who were refractory to chemotherapy showed a potentiated p-Stat3/p-Stat5 signaling response, suggesting the role of alternate oncogenic pathways in their leukemia [5]. In a recent report, SCNP analysis of a separate cohort of AML samples taken at diagnosis was used to describe correlations between an expanded panel of intracellular signaling pathways with clinical response to induction therapy [28].

**Figure 1:**
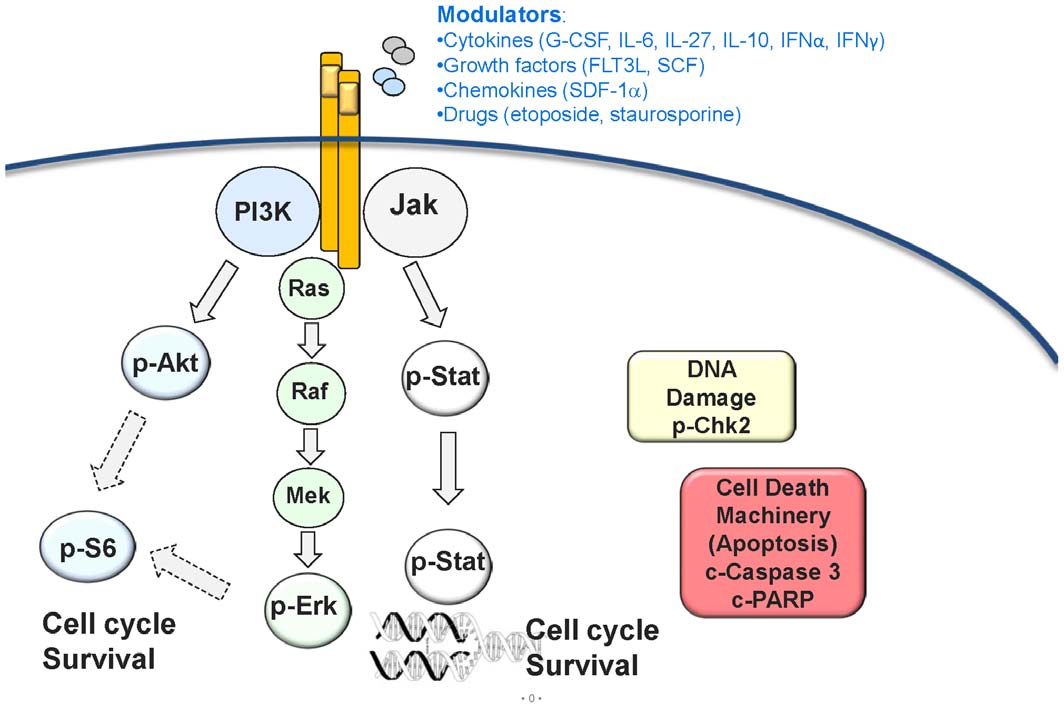
Signaling, DDR and apoptosis pathways evaluated in AML blasts. Jak/Stat & PI3K growth and survival pathways were measured in response to modulation with cytokines, interleukins, growth factors and chemokines in AML samples. DDR and apoptotic machinery were measured after *in vitro* exposure of AML samples to etoposide and staurosporine.

In this report, we describe a detailed biological characterization of the same AML samples (used in the clinical study) based on the activities of their intracellular signaling pathways and their relationships to each other. In addition to Jak/Stat signaling, other pathways were included to provide a more expansive view of aberrant myeloid biology in AML including alternative pathways by which cells evade apoptosis ([29], Figure 1). Analysis of Jak/Stat, PI3K, DNA damage response (DDR) and apoptosis pathway activities characterized biologically distinct patient-specific profiles, even within cytogenetically and cell surface uniform patient subgroups. Thus, while AML is known to be clinically heterogeneous, the biology described in this study shows that it is reducible to a limited number of intracellular signaling pathways highlighting survival pathways, DDR and their link to apoptosis.

## Materials and Methods

### Ethics Statement

This study was conducted according to the principles expressed in the Declaration of Helsinki. The study was approved by the Ethics Review Board of the University Health Network, University of Toronto hospital. All patients provided written informed consent for the collection of samples and subsequent analysis.

### Patient Samples

Thirty four diagnostic cryopreserved non-M3 AML peripheral blood mononuclear cell (PBMC) samples taken from patients within the University Health Network, University of Toronto were used in this study. Patient characteristics are shown in Table S1. All patients had given their consent for the collection and use of samples for institutional review board (IRB) approved research purposes. All clinical data were de-identified in compliance with Health Insurance Portability and Accountability Act (HIPPA) regulations. Induction chemotherapy consisted of at least one cycle of cytarabine-based standard induction therapy (daunorubicin 60 mg/m^2^ ×3 days, cytarabine 100–200 mg/m^2^ continuous infusion ×7 days) [30] and responses were measured after one cycle of induction therapy. The criteria for reporting an outcome as complete response (CR) were defined as follows: marrow aspirate with less than 5% leukemic blasts, neutrophils greater than 1,000/mcL, platelets greater than 100,000/mcL and no residual evidence of extramedullary disease. Leukemic samples obtained from patients who did not meet the criteria for CR were considered non-complete response (NR). Patient outcomes were blinded until completion of experiment and acquisition of data.

### Cell Preparation

Samples were thawed at 37°C and resuspended in RPMI 1640 1% FBS. Amine aqua (Invitrogen) was used to determine cell viability according to the manufacturer's protocol. Cells were resuspended in RPMI 1% FBS, divided into aliquots of 100,000 cells/condition and rested for 2 hours at 37°C. For apoptosis assays, cells were incubated with staurosporine or etoposide for 6 hours and 24 hours respectively. These time points were chosen based on data generated from foundational experiments in AML cell lines and myeloid cells gated from healthy PBMCs, such that only some, but not all cells underwent apoptosis. For all other assays, cells were incubated at 37°C with modulators as described in Tables S2A and S3 for 3–15 minutes. Since the amount of sample was limiting the decision was taken to measure signaling in response to multiple modulators, rather than multiple time points with fewer modulators. The latter is planned for future studies. Cells were subsequently fixed with paraformaldehyde and permeabilized with 100% ice-cold methanol as previously described [31]–[33]. For apoptosis assays, cells were re-stained with amine aqua before fixation and permeabilization. Cell lines U937 and GDM-1 as well as PBMC and BMMC samples from healthy donors were included as controls to ensure quality of assay performance.

### Flow cytometry determinations of survival, proliferation, DDR and apoptotic pathways

Methanol permeabilized cells were washed with PBS 0.5% BSA 0.05% NaN_3_ (FACS Buffer), pelleted, and incubated with antibodies to cell surface antigens CD33 and CD45 [31]–[33]. The latter allowed leukemic blasts to be delineated and distinguished from non-myeloid cells. Concurrently with surface marker antibodies cells were incubated with antibodies recognizing intracellular signaling molecules within survival, proliferation, DDR or apoptosis pathways (Tables S2 and S3). Antibodies were used at saturating concentrations, pre-determined from titrations in cell lines or in the myeloid cell population of cryopreserved PBMCs from healthy donors. Antibody specificity was determined by use of isotype controls or, in the case of phospho-antibodies, pre-blocking with the phospho-peptide epitope against which the antibodies were generated (data not shown).

### Data Acquisition and Cytometry Analysis

Data was acquired using FACS DIVA software (BD) on an LSR II Flow Cytometer (BD) equipped with a high throughput sampler (HTS) and gated using FlowJo (Treestar). For all analyses, dead cells and debris were excluded by forward scatter (FSC), side scatter (SSC) and amine aqua viability dye. Leukemic cells were identified as cells that lacked the characteristics of mature lymphocytes (CD45^++^ CD33^−^), and that fit the CD45 and CD33 vs. right angle light scatter characteristics consistent with myeloid leukemia cells [33], Figure S1).

### Metrics

Several metrics were developed to measure the level of activation of an intracellular signaling protein (See Methods & Figure S2). The “Basal” metric quantifies the level of activation for a protein in its resting state. The “fold” metric is the level of activation of a signaling molecule after treatment with a modulator compared to its level of activation its basal state. The “Total” metric measures the total magnitude of an activated protein and includes the level of basal and induced protein activation. It is more relevant for pathway measurements regulated by activity thresholds. For apoptosis assays, the percentage of cells that undergo cell death in response to an agent was measured with the amine aqua viability dye and with antibodies against cleaved PARP [28].

The following metrics were applied to all nodes, (See Figure S2):

**Basal**: log_2_ [Median fluorescence intensity (MFI)(Unmodulated)/MFI(Autofluorescence)]

**Fold**: log_2_ [MFI(Modulated)/MFI(Unmodulated)]

**Total**: log_2_ [MFI(Modulated)/MFI(Autofluorescence)]

Apoptosis was calculated as the percentage of induced c-PARP^+^ and/or Aqua^+^ cells:

% Apoptotic  =  % c-PARP^+^ and/or Aqua^+^.

**Apoptosis**: % Apoptotic (Modulated) - % Apoptotic (Unmodulated) (See Figure S2).

Samples displaying high basal apoptosis (greater than 60%) after overnight incubation in media were excluded from the apoptosis analyses. Analysis of p-Chk2 induction was performed in non-apoptotic c-PARP^−^ cells.

### Principle Component analysis (PCA)

PCA was performed as described [34] for Jak/Stat and PI3K nodes using both “Fold and “Total” metrics of induced pathway activity along with the corresponding basal nodes.

### Choice of Node/Metrics for PCA

All node/metrics were independently tested by univariate analysis for their ability to classify patients based on their disease response to standard induction therapy. Jak/Stat and PI3K nodes that stratified clinical CR and NR patients (area under the curve of the receiver operator characteristic (AUC_ROC_) >0.6 and p<.05) are listed in Table S4 and were used for principle component analyses and for selecting examples of the node/metrics that were used to construct the heat-maps.

### SCNP Assay Terminology

The term “signaling node” is used to refer to a proteomic readout in the presence or absence of a modulator. For example, the response to G-CSF stimulation can be measured using p-Stat5 as readout. That signaling node is designated “G-CSF→p-Stat5”. Several metrics (normalized assay readouts summarized below and defined in Figure S2) are applied to interpret the biology of each signaling node and are noted following the node e.g. “G-CSF→p-Stat5 | Fold”, “G-CSF→p-Stat5 | Total” or “p-Stat5 | Basal”.

## Results

Given the prosurvival role of Jak/Stat and PI3K signaling in cancer, functional performance testing via SCNP analysis of these pathways was carried out in AML blasts after their exposure to a panel of modulators known to play a role in myeloid biology through engaging these pathways [24]–[25], [35]–[38].

### Jak/Stat pathway activity

**Figure 2:**
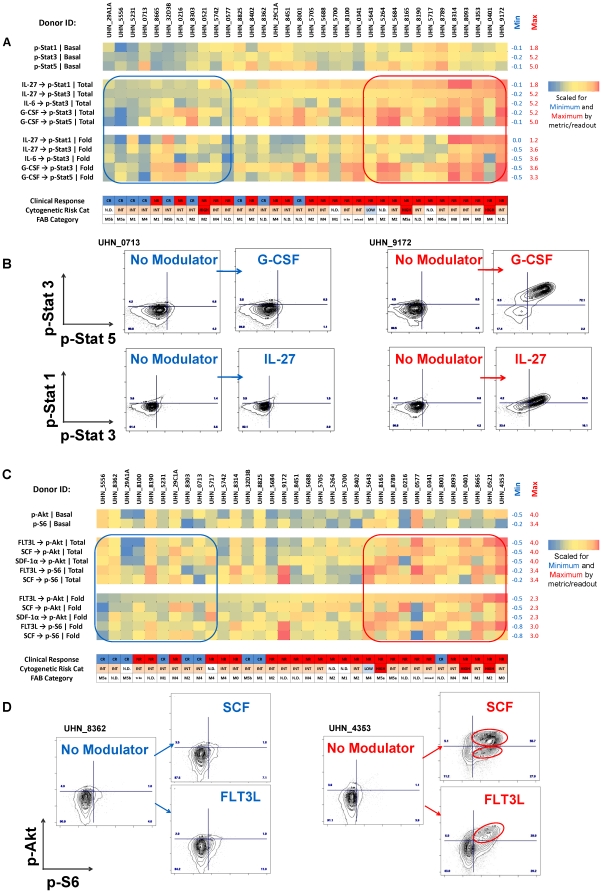
Induced Jak/Stat and PI3K/S6 signaling varies among AML samples. Pathway activity is represented by heat-maps. Blue: Low Signaling, Red: High Signaling, scaled for an individual readout/metric. (A) Jak/Stat pathway activity was measured after treatment of AML samples with IL-27, IL-6, and G-CSF. Heat map of Jak/Stat signaling responses, where each row represents a specific readout and metric such as: p-Stat1 | Basal, IL-27 → p-Stat1 | Total. The samples have been ordered according to IL-27 → p-Stat3 | Total, since this node had the highest AUC and p-value for stratifying CRs from NRs [28]. Cytogenetic risk and FAB categories are noted for each sample. (B) Exemplary 2D flow plots showing signaling responses to G-CSF or IL-27 treatment. (C) PI3K pathway activity was measured after treatment of AML samples with FLT3L, SCF and SDF-1α. Heat map of PI3K signaling responses, where each row represents a specific readout and metric. Patient samples have been ordered according to FLT3L → p-Akt | Fold responses. (D) Exemplary 2D Flow plots showing signaling responses to FLT3L or SCF treatment.

To assess the activity and inducibility of the Jak/Stat pathway, samples were treated with G-CSF, IL-6, IL-27, IL-10, IFNα and IFNγ, known to activate the Jak/Stat pathway. AML samples were characterized by the magnitude of their basal Jak/Stat pathway activity as well as by the induced responses (Fold metric) and total level of Jak/Stat pathway activation (Total metric) with examples shown by the heat-maps in Figure 2A). In many, but not all cases, the latter two metrics used paralleled each other. Low or absent levels of induced phosphorylation of Stat1, Stat3 and Stat5 proteins were associated with gated AML blasts from CR patients exemplified by the 2D flow plots shown for responses of sample UHN_0713 to G-CSF and IL-27 (Figure 2B). In contrast, potentiated Jak/Stat signaling was observed as well as increased pathway activity in cells taken from patients whose leukemia was non-responsive to induction chemotherapy, as exemplified in a 2D flow plot for myeloid-gated cells for sample UHN_9172 (Figure 2B). In most NR patient samples Jak/Stat signaling was elevated in a cell subpopulation in response to multiple cytokines (right hand side of heat-map in Figure 2A), whereas cells of most CR patients were largely non-responsive (grouped towards the left hand side of the heat-map in Figure 2A). IL-27 and IL-6-mediated-phosphorylation of Stat3 were closely correlated, as would be expected for two cytokines sharing the gp130 common signal transduction receptor subunit [39]–[41].

### PI3K pathway activity

A second major survival pathway interrogated in this study was PI3K, known to play a role in most cancers [24]–[25], [42]. Converging signals from the PI3K/mTor and Ras/Erk pathways result in phosphorylation of ribosomal protein S6 which correlates with increased protein translation of mRNA transcripts that encode proliferation and survival promoting proteins [43]–[44].

Analogously to activation of the Jak/Stat pathway, application of known activators of the PI3K pathway including FLT3L, SCF and SDF-1α broadly grouped AML samples by the magnitude of their signal transduction responses (Fold metric) and overall pathway activity (Total metric) represented by measurements of p-Akt and p-S6 (Figure 2C). In the same manner that low levels of modulated Jak/Stat responses and Jak/Stat pathway activity were seen in leukemic cells from CR patients (Figure 2A), samples in which p-Akt/p-S6 signaling was low or absent (exemplified by the 2D flow plot for sample UHN_8362 in Figure 2D) were also associated with clinical responsiveness to chemotherapy. Additionally, in the same manner that high levels of induced Jak/Stat responses and high levels of Jak/Stat pathway activity were seen in leukemic cells from NR patients, elevated PI3K pathway responses were also associated with clinical non-response to chemotherapy (exemplified by the 2D flow plot for sample UHN_4353 in Figure 2D). Importantly, no associations could be made between cytogenetic risk category and the French American British category (FAB) [18] within these signaling responses (Figure 2A and 2C).

### Correlated measures of induced JAK/STAT and PI3K signaling reveals AML blasts with distinct pathway responses

**Figure 3:**
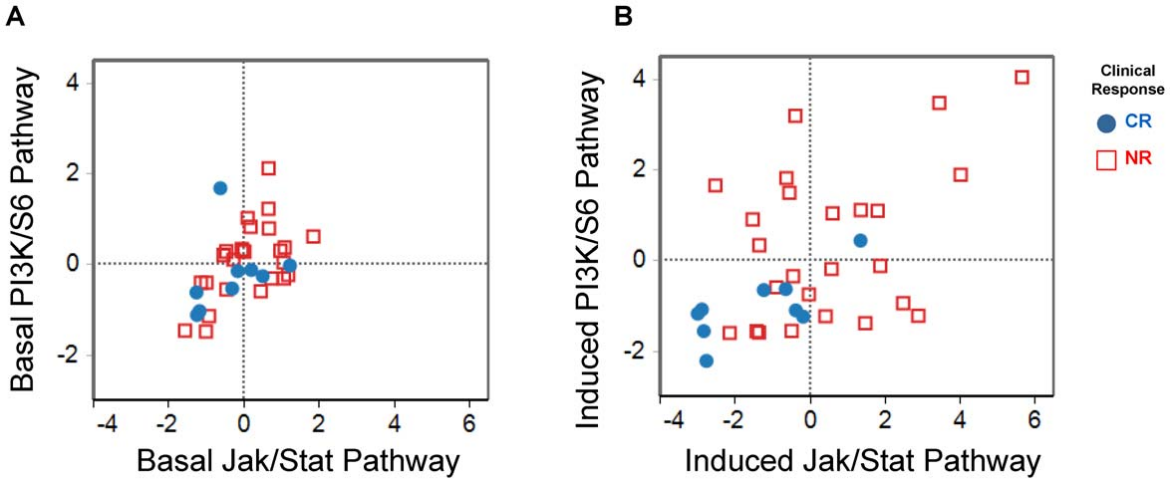
Pathway analysis of induced Jak/Stat and PI3K activity reveals distinct profiles. Principal Component Analysis (PCA) of NR samples (unfilled red squares) compared to CR samples (filled blue circles) for Jak/Stat and PI3K signaling. The metrics used in the analysis were “basal”, “fold” and “total”. (A) Basal signaling: basal metric values for p-Stat1, p-Stat3, p-Stat5 and p-Stat6 were chosen to represent Jak/Stat pathway signaling, while the basal values for p-Erk, p-S6 and p-Akt were chosen to represent the PI3K pathway. The first principal component for the Jak/Stat pathway explained 85% of the variance while the one for the PI3K pathway explained 71% of the variance. (B) Induced signaling: the metrics used in the analysis were “fold” and “total” metric for the same intra-cellular readouts used for the PCA of basal signaling. The percentage of variance explained by the first principal component for induced Jak/Stat signaling was 66% and for induced PI3K signaling it was 46%. The PCA reveals a heterogeneity of profiles for NR samples when treated with modulators which is not seen in the PCA of untreated samples in their basal state.

In order to understand the activation state for both the Jak/Stat and PI3K pathway activities in individual AML samples, principal component analyses were performed for each pathway in its basal state as well as its functionally potentiated state combining readouts from multiple modulators known to activate each pathway [34]. Induced pathway activity, rather than basal pathway activity, could more readily reveal distinct patient-specific functional response patterns (Figure 3). The data revealed that there were multiple response profiles in individual AML samples such that activity was high or low for both pathways or high for one and low for the other pathway (Figure 3B). Interestingly, although there are only 9 samples from CR patients (Figure 3, filled blue circles), a low signaling capacity in both the Jak/Stat and PI3K/S6 pathways was associated with clinical response to chemotherapy. By contrast, augmented signaling responses from one or both the Jak/Stat and PI3K pathways were observed in most samples from chemotherapy refractory patients (Figure 3, unfilled red squares). Furthermore, in a sub-group of NR AML blast samples (Figure 3, red squares in the lower left hand quadrant) low level signaling responses in both Jak/Stat and PI3K pathways were seen, suggesting that other pathways could be contributing to clinical refractoriness to chemotherapy. Taken together, these data suggest that activation of the PI3K and Jak/Stat pathways (or pathways or cell states which are more proximally causal, but correlated with the activities seen via PI3K and Jak/Stat), might oppose response to chemotherapy.

### Measurements of DDR and apoptosis with *in vitro* exposure to etoposide and staurosporine

**Figure 4:**
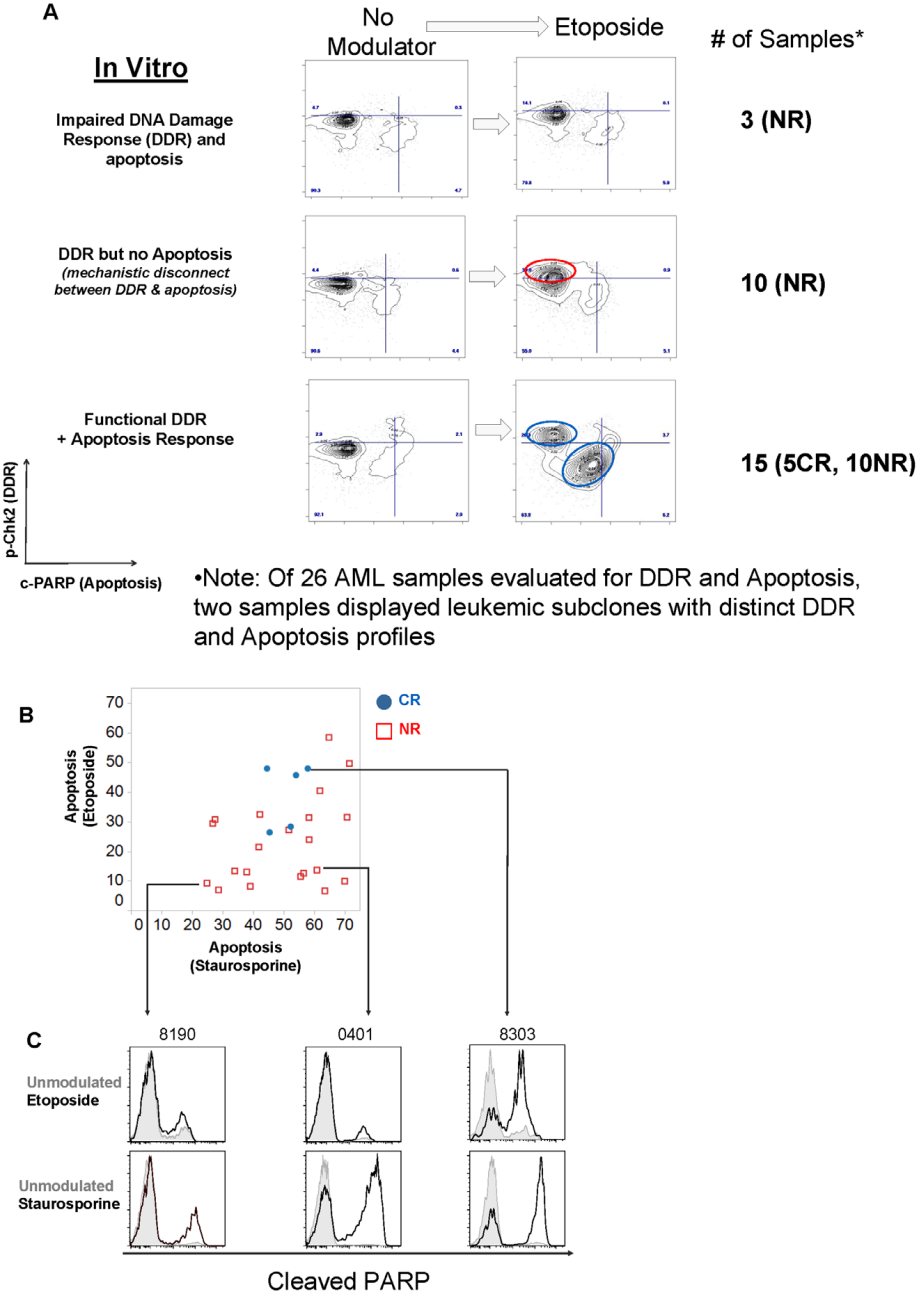
Distinct *in vitro* DDR and apoptosis response profiles identified in AML samples. DDR and apoptosis were measured by p-Chk2 and Aqua positivity with cleaved PARP respectively. (A) Three DDR/apoptosis profiles identified in AML samples after 24h *in vitro* exposure to etoposide. (B) Scatter plots to show correlations between apoptosis response of AML samples to etoposide and staurosporine. (C) Exemplary histograms showing cleaved PARP readouts for different responses to etoposide and staurosporine.

In addition to the major role played by survival pathways in AML, the DDR pathway is also critical, as DNA damaging agents are at the core of most AML treatment regimens [30]. Many agents used to treat AML promote double stranded DNA breaks resulting in activation of cell cycle checkpoints which suspend passage through the cell cycle and allow time for DNA repair [45]–[48]. If the resultant damage cannot be repaired then cells are triggered to undergo apoptosis. In this study, DDR and apoptosis were measured simultaneously in AML blasts, post exposure to etoposide, using antibodies against phospho-threonine 68 on the checkpoint kinase, Chk2, and against cleaved PARP respectively. Etoposide, is a topoisomerase ll inhibitor, that induces DNA double-stranded breaks [45], [49]. Three distinct responses exemplified by the 2D flow plots in Figure 4A were observed: (1) AML blasts with a defective DDR and failure to undergo apoptosis (2) AML blasts with proficient DDR and failure to undergo apoptosis (3) AML blasts with proficiency in both DDR and apoptosis (Figure 4A). Notably, all CR samples were exemplified by the third profile whereas NR samples were exemplified by all three response profiles.

To determine whether the apoptotic machinery was intact in etoposide profiles 1) and 2), cells were exposed to staurosporine, a multi-kinase inhibitor that promotes apoptosis through G1 arrest without the participation of DDR as described for etoposide [50]. Apoptosis was measured using fluorochrome-conjugated antibodies against cleaved caspase 3 and cleaved PARP in addition to a cell viability dye. The data shown are with cleaved PARP and the cell viability dye. Cleaved caspase 3 closely mirrored the cleaved PARP data but in the interests of brevity are not shown. Apoptosis responses were evaluable in 26/33 of the AML samples (See Methods) and are depicted by the scatter plot in Figure 4B.

A comparison of etoposide versus staurosporine-mediated apoptosis showed different response profiles for each sample at the timepoint chosen for the assay (Figure 4B). Many samples in which the majority of the leukemic blasts were refractory to etoposide were responsive to staurosporine. This implies that the apoptotic machinery *per se* was intact in these cells and that the resultant refractory response to etoposide could be the result of ineffective communication between the machinery of the DDR with that of apoptosis (exemplified by sample UHN_0401, Figure 4C). Other categories of response shown are relative refractoriness to both agents (exemplified by sample UHN_8190, Figure 4C) or responsiveness to both agents (exemplified by sample UHN_8303, Figure 4C). Furthermore, the plot shows percentage of cells within an AML sample undergoing apoptosis and for no sample was this value 100% at the timepoints chosen in this study (Figure 4B). This indicates that within an AML sample there are blast cell subsets with different sensitivities to each agent.

In spite of the small and biased (for clinical response) sample set, all samples with blast subsets refractory to *in vitro* etoposide exposure, regardless of their staurosporine response, were derived from the NR patient sample subgroup (Figure 4B). Apoptosis responses identified all CR patients as apoptosis competent to both agents. However, a negative apoptotic response could not predict all NR patients, underscoring the fact that *in vitro* responses alone to apoptosis stimulating agents are only part of the equation that describes a clinical outcome (Figure 4B).

### Associations Between *in vitro* apoptosis profiles with Jak/Stat and PI3K pathway activity

**Figure 5:**
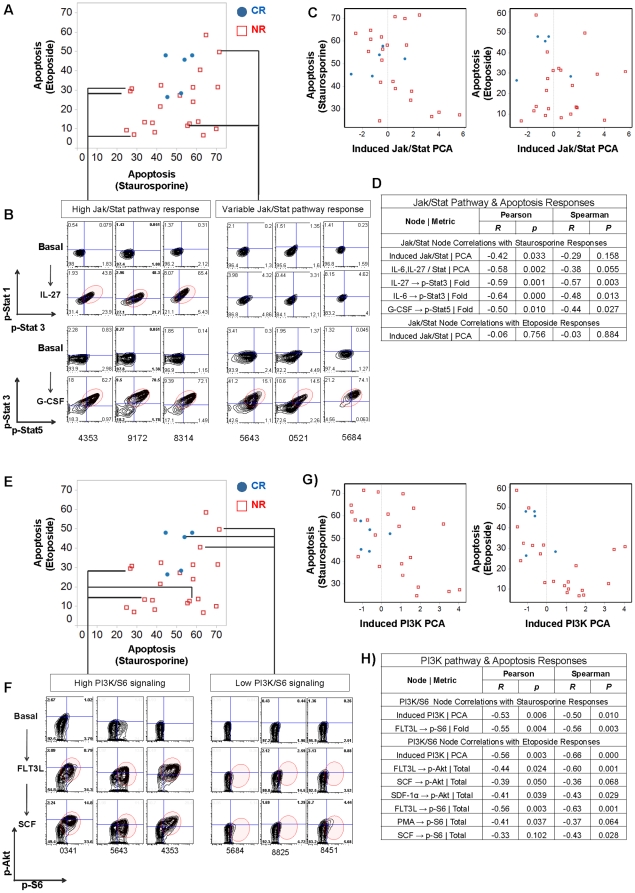
Correlations between Jak/Stat and PI3K pathway activity with apoptotic responses. Scatter plots were generated to determine the correlations between pathway activity (PCA analysis of modulated Jak/Stat and PI3K signaling) with apoptosis responses to etoposide and staurosporine. Filled blue circles denote samples from CR patients and unfilled red squares denote samples from NR patients. (A) Scatter plot to show correlations between response to etoposide and staurosporine with exemplary 2D flow plots (B) showing Jak/Stat pathway responses after treatment with a specific modulator. (C) Scatter plots showing correlations between PCA of Jak/Stat pathway activity with either etoposide or staurosporine. (D) Pearson and Spearman correlation coefficients and probabilities show a general inverse correlation between Jak/Stat pathway activity with apoptotic responsiveness to staurosporine but not to etoposide with outliers shown in the plot. (E) Scatter plot to show correlations between response to etoposide and staurosporine with exemplary 2D flow plots (F) showing PI3K pathway responses after treatment with a specific modulator. (G) Scatter plots showing correlations between PCA of PI3K pathway activity with either etoposide or staurosporine. (H) Pearson and Spearman correlation coefficients and probabilities show a general inverse correlation between PI3K pathway activity with apoptotic responsiveness to etoposide and staurosporine with outliers shown in the plot.

To relate proliferation and survival signaling to *in vitro* apoptotic potential, the Jak/Stat and PI3K pathway activities observed in leukemic samples (Figures 2 and 3) were analyzed in the context of the *in vitro* apoptotic responses described above (Figure 4B). Overall, Jak/Stat signaling responses were of variable magnitude for samples with relatively low or high responsiveness to etoposide (Figure 5A, 5B, 5C). This was also true for samples that were sensitive to staurosporine (Figure 5A, 5B, samples UHN_5643, UHN_0521, UHN_5684 and 5C). However, for four samples with the lowest relative response (relative refractoriness) to staurosporine, Jak/Stat pathway responses were augmented (Figure 5A, 5B, samples UHN_4353, UHN_9172, UHN_8314 and 5C). Pearson and Spearman coefficients showed that there was in general a statistically significant negative correlation between staurosporine induced apoptosis and Jak/Stat signaling in this AML sample set, with outliers clearly apparent (Figure 5D). Statistical significance was found for the Jak/Stat PCA value with even greater statistical significance observed for individual nodes such as IL-6 or IL-27 induced Stat signaling (Figure 5D). Pearson and Spearman coefficients revealed a lack of correlation for Jak/Stat signaling with etoposide response (Figure 5D).

Consistent with their roles in survival there was an inverse correlation between levels of growth factor (SCF and FLT3L) and chemokine (SDF-1α)-mediated-p-Akt and p-S6 signaling and *in vitro* apoptotic response. This relationship was statistically significant as shown by computing the Pearson and Spearman correlation coefficients (Figure 5G, 5H). Thus, induced PI3K pathway signaling tended to be lower for samples that were apoptosis proficient to both etoposide and staurosporine (Figure 5E, 5F, samples UHN_5684, UHN_8825 and UHN_8451 and 5G). Greater induced p-Akt and p-S6 levels were observed in samples refractory to staurosporine and/or etoposide (Figure 5E, 5F, samples UHN_0341, UHN_5643 and UHN_4353, 5G).

When taken together, trends for apoptosis, Jak/Stat and PI3K pathway activities (Figures 3, 4, and 5) and clinical outcomes (Table S1) suggest that there are limited number of pathway profiles associated with CR patients, whereas in NR patients many different pathway mechanisms may have evolved for the leukemia to be refractory to chemotherapy. Notably, all samples from CR patients had blast cell subsets that were sensitive to *in vitro* staurosporine and etoposide-mediated apoptosis and in general had low Jak/Stat and PI3K pathway responses (Figure 3B, 5C, 5G). Most clinical NR samples that were competent to undergo *in vitro* apoptosis had an absent or low PI3K response (Figure 3B, 5G), suggesting that other pathways could be contributing to clinical refractoriness to chemotherapy. All other NR samples were refractory to *in vitro* etoposide and/or staurosporine exposure with different degrees of elevated Jak/Stat and/or PI3K pathway activation.

### Leukemic samples with two blast populations that each harbor cell subpopulations with distinct pathway profiles

**Figure 6:**
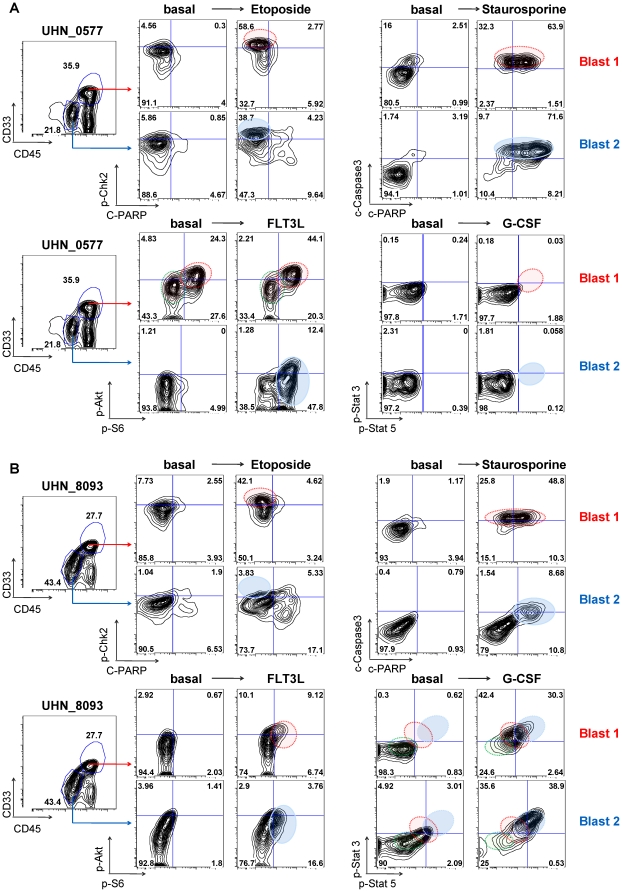
Distinct blast subsets defined by surface phenotype and signaling in an individual sample. Samples (UHN_0577) (A) and UHN_8093 (B) are comprised of Blast 1 and Blast 2 populations as shown by CD33 and CD45 surface marker expression. Each blast population displays distinct biological properties in leukemic sub-clones as exemplified by responses to etoposide, staurosporine, FLT3L, and G-CSF. (A) SCNP analysis of sample UHN_0577, (B) SCNP analysis of samples UHN_8093.

Analysis of CD33 and CD45 surface expression of all samples within this AML cohort defined three patient samples with two distinguishable leukemic cell subpopulations, referred to as Blast 1 and Blast 2. In all cases, Blast 1 was defined as a cell subset with higher CD33 and CD45 levels, whereas Blast 2 cells had lower levels of these surface proteins. Given the distinct signaling profiles identified for cell subsets within samples harboring only one myeloid blast population as defined by CD33 and CD45 expression, in the preceding data of this study, it seemed likely that samples harboring two myeloid blast populations could harbor distinct signaling profiles. Although seemingly homogeneous by surface phenotype, SCNP revealed distinguishable signaling responses within individual cells in each blast population measured simultaneously. In what follows, signaling and apoptosis data from two of the three samples, both from NR patients, are shown in Figure 6. Blast populations 1 and 2 from sample UHN_0577 were refractory to etoposide-mediated apoptosis although both populations exhibited DDR, albeit to different magnitudes as seen by the frequencies of blasts with increased phosphorylation of p-Chk2 (Figure 6A, Table S5). Exposure of the samples to staurosporine revealed that the apoptotic machinery was intact in both blast populations suggesting that etoposide refractoriness was the result of disabled communication between DDR and the apoptotic machinery. Comparison of each blast subset for its response to G-CSF revealed minimal increases in p-Stat3 and p-Stat5. However, inspection of the PI3K pathway revealed that Blast 1, but not Blast 2 had two discernible blast cell subsets with different levels of p-Akt and p-S6 in the basal state. Blast 2 had only one “low” level p-Akt and p-S6 blast cell subset. Furthermore, in Blast 1, FLT3L was able to induce both p-Akt and p-S6 signaling in the “low level” basal population. In contrast, for Blast 2 the predominant response to FLT3L was an increase in p-S6 alone. Using the metric of “total” as a measure of overall pathway activity (Table S5), there was greater overall pathway activity for Blast 1 than for Blast 2 in both the basal and FLT3L-potentiated states reflecting significant contributions of both basal and evoked signaling responses.

The two blast populations in sample UHN_8093 were both refractory to etoposide possibly through different mechanisms since there was a greater p-Chk2 response in Blast 1 and a much reduced DDR in Blast 2 (Figure 6B). Blast 1 was very responsive to staurosporine which indicated that the apoptotic machinery is intact and that the etoposide refractoriness in Blast 1 could be accounted for by failure of DDR to communicate with the apoptotic machinery. In contrast, Blast 2 was refractory to staurosporine-mediated apoptosis. Notably, in Blast 2 G-CSF mediated greater increases in phosphorylated Stat3 and Stat5 compared to the increases seen in Blast 1. This was reflected by both the “fold” and “total” metrics (Figure 6B, Table S5). Inspection of PI3K pathway activity revealed that only a small blast cell subset responded to FLT3L treatment with the majority of cells remaining unresponsive. These data suggest that the higher activity seen for the Jak/Stat pathway for Blast 2 may account for its refractoriness to *in vitro* apoptosis and non-response in the clinic consistent with the data in Figure 5.

## Discussion

The current study was designed to determine whether individual AML samples can be characterized based on *in vitro* functional performance tests using SCNP to measure survival pathways, DDR and *in vitro* apoptosis. The major findings were that: (i) an individual sample can be comprised of leukemic blast subsets with distinct Jak/Stat, PI3K, DDR and apoptosis pathway responses, (ii) exposure of samples to modulators allowed these pathway responses to be revealed, (iii) PI3K pathway activity was high in most samples that were refractory to apoptosis-inducing agents *in vitro*, (iv) Jak/Stat pathway activity was high in samples refractory to staurosporine but only in some samples refractory to etoposide, (v) *in vitro* DDR and apoptosis profiles were variable in leukemic blasts between different samples and also within the same sample and (vi) SCNP of the pathways chosen reveal a restricted number of profiles for AML blasts from CR patients and multiple profiles for AML blasts from NR patients.

Thus, responders to chemotherapy demonstrated little variation in the signaling potential of the pathways evaluated (that is, cells remained relatively unperturbed by environmental stimuli applied). As such, in the CR samples both the potentiated responses to myeloid activators of the Jak/Stat and PI3K pathways, as well as “basal” pathway activity tended to be low (Figure 3) whereas DDR with subsequent apoptosis was robust after *in vitro* etoposide exposure (Figure 4A). By contrast, robust Jak/Stat and PI3K responses were revealed in most NR samples (Figure 3). These data are consistent with, and expand upon previous findings linking functional alterations in Jak/Stat signal transduction with poor response to chemotherapy in AML patients [5]. In addition, all samples with impaired DDR or proficient DDR without subsequent apoptosis were NRs (Figure 4A). A subset of NR samples were competent to undergo *in vitro* apoptosis and had low PI3K and Jak/Stat pathway responses suggesting that in these samples alternative pathways could be contributing to clinical refractoriness to chemotherapy (Figure 3). The data suggest that while DDR, Jak/Stat, and PI3K pathways might serve as useful indicators of the biological underpinnings of therapeutic responses, additional inquiry into alternative pathways might be required to more fully complete the characterization of response.

This study used 34 diagnostic PBMC samples taken from patients for which clinical outcomes were blinded. However, the sample set was unintentionally biased with samples predominantly from NR, female patients of younger age with intermediate cytogenetics (Table S1). In spite of these limitations, univariate analysis of this sample set and an independent sample set (with 57 CR samples out of a total of 88 samples) from a separate institution revealed common nodes for CR and NR stratification suggesting that survival, DDR and apoptosis pathways may be relevant ways to characterize AML disease subtypes with further follow-up warranted in additional AML sample cohorts [28].

The proliferative and survival properties of the Jak/Stat and PI3K pathways most likely play a central role in AML leukemogenesis as well as in refractoriness and resistance to clinically used DNA damaging agents. For instance, Stat transcription factors are known to play a critical role in normal and leukemic hematopoiesis targeting transcription of genes involved in proliferation, survival and differentiation [51]–[54]. Receptors that signal through Stat3 and Stat5 are present on AML blasts where they can be activated by a wide variety of growth factors, interleukins and cytokines [55]. Furthermore, in a recent study, the level of Stat5 transcriptional activity was shown to regulate the balance between proliferation and differentiation in hematopoietic stem cells/progenitor cells by activating specific genes associated with these processes [56]. The same group showed that high levels of Stat5 activity disrupted myelopoiesis. Additionally, although frequently cited as anti-survival, recent reports have described a wide variety of roles for Stat1 including cell proliferation, survival, apoptosis, and differentiation [57]. Here, Stat1 is activated in some samples simultaneously with Stat3 and Stat5, although its precise functional role warrants further investigation. In the current study, CR samples showed low or absent Jak/Stat responses and a subset of NR samples showed high magnitudes of Jak/Stat responses while the remaining NRs displayed a continuum of responses. (Figure 2A, B). These data suggest that certain levels of Stat activity may be necessary to generate the appropriate transcriptional program necessary for maintaining a particular clonal state of an AML blast cell subset.

In addition, deregulation of the PI3K/mTor signaling pathway has been described in 50–80% of AML cases contributing to the survival and proliferation of AML blast cells [58]–[59]. Many causes for pathway deregulation have been cited such as activating mutations in FLT3 and Kit receptors, overexpression of the PI3K class 1A p110δ isoform as well as gain of function mutations in N- and K-Ras [37], [60]–[63]. In this study, PI3K pathway activity was determined by measuring levels of p-Akt and p-S6 ribosomal protein as pathway readouts in response to myeloid modulators, FLT3L, SCF and SDF-1α. Consistent with its role in cancer cell survival [24], potentiated levels of p-Akt and p-S6 were lower in CRs and elevated in clinical NRs, although the two clinical categories were not mutually exclusive since several NR samples had low potentiated PI3K pathway activity (Figure 2C, D).

Moreover, alternative mechanisms of refractoriness could arise from increased DDR, failure to undergo DDR and/or inoperative communication between DDR and apoptosis [46]–[47]. For a response to a DNA damaging agent, DNA lesions recruit multi-protein DNA damage sensor complexes that associate with DNA damage transducer proteins such as ataxia telangectasia mutated (ATM), a kinase which upon activation phosphorylates Thr68 (T68) of the checkpoint kinase Chk2. The resultant delay in cell cycle progression provides cells with a chance to repair the DNA damage. If repair fails, cells undergo apoptosis [46]–[48]. In this study three DDR/apoptosis profiles distinguished AML samples. In the first, minimal p-Chk2 response was observed and consequently no apoptotic response. In the second profile, there seemed to be a failure for DDR to translate into apoptosis and in the third, DDR, apoptosis and their communication was intact. Notably, all clinical responsive patients fell into this latter category. Further sample cohorts are needed to see whether this association between *in vitro* apoptotic sensitivity and clinical response holds, potentially providing a valuable means for predicting clinical outcomes.

The robust activation of two major survival pathways shown in a subset of AML samples provided a rationale for evaluating apoptotic proficiency in this sample cohort. *In vitro* exposure of samples to etoposide and staurosporine [49]–[50], two agents that induce apoptosis by different mechanisms, identified distinct blast subsets with different responses to each agent between individual samples and also within the same sample (Figure 4B, 4C, Figure 6). Samples sensitive to both agents were taken from CR patients. However, this apoptotic proficiency was also observed in some NR patient samples. There are several explanations to account for the unexpected *in vitro* apoptotic response of NR samples, principally that the *in vitro* apoptotic responses were not measured with the drugs used clinically (ara-C/daunorubicin) by which the NRs were categorized. Further, although etoposide, ara-C and daunorubicin all induce DNA damage, they have different mechanisms of action and are substrates for different transporters [64]–[66] and thus might not mimic the *in vivo* responses. It is also possible that the AML biology characterized for these samples is not represented by clinical definitions of NR and CR. Furthermore, in all cases, only a fraction of cells undergo apoptosis and the phenotype of the non-responding cells may account for the apparent disconnect between apoptosis seen *in vitro* versus the clinical NR.

In order to understand whether there was a link between signaling by survival pathways and *in vitro* apoptotic responses, correlations (Figure 5) were computed from the data depicted in Figures 3 and 4B. Notably, when evaluated for Jak/Stat and PI3K pathway activity, most samples refractory *in vitro* to either or both etoposide and staurosporine had a cell subset that displayed potentiated PI3K signaling (Figure 5E –5H). In contrast, samples refractory to staurosporine displayed elevated Jak/Stat pathway activity whereas there were variable levels of Jak/Stat pathway activity across a range of etoposide induced responses (Figure 5A –5D). Given the fine balance between levels of p-Stat5 that, via a transcriptional program *in vivo*, regulate blast cell proliferation versus disruption of differentiation, the *in vitro* experimental conditions utilized here may not have allowed these more subtle changes to be observed between Stat activity and DDR induced apoptosis [56]. It is very likely that these two common survival pathways are playing a major role in conferring refractoriness to chemotherapy, but that alternative, as yet unrevealed, pathways also make a contribution.

Several AML samples within this cohort had two blast cell populations discernible by their surface phenotype suggestive of cell populations representing different stages of differentiation. Of the two samples described in this manuscript, SCNP revealed that each blast cell population had its own distinct signaling and apoptosis profiles (Figure 6A, 6B). Given the opportunity to apply SCNP assays to samples taken over time from the same patient it may be possible to determine which blast population confers refractoriness to chemotherapy.

Further correlations to defined genetic abnormalities driving these signaling observations could underscore their potential roles in driving AML disease; such as analysis of intracellular signaling pathways in the context of FLT3 mutational status (manuscript in preparation). The output from such studies could be to guide the choice of available investigational and approved agents to provide benefit for those AML patients refractory to current chemotherapy regimens [21], [67]–[68].

## Supporting Information

Figure S1 Sequential gating scheme shown by 2D flow plots A) Non-cellular debris was excluded using a FSC and SSC gate. B) Non-viable cells were excluded with a SSC and aqua viability dye gate. C) Leukemic blasts were gated by CD45, CD33.
(2.10 MB TIF)

Figure S2 Definition of Metrics A) Summary of metrics used and the role each has in determining a different measure of signaling biology. Median Fluorescence Intensities (MFI) were calculated for leukemic blast cells under each condition and used to compute the metrics that represented protein expression, signaling and apoptosis, as described in Materials and Methods. Apoptosis was measured by levels of c-caspase 3, c-PARP as well as cells that were positive for the aqua viability dye. Measurements for cleaved caspase 3 were found to be correlated with cleaved PARP, but this was not the case for cleaved PARP and aqua. Therefore apoptosis metrics incorporated measurements for aqua and cleaved PARP to quantify cells that became positive for c-PARP alone or for both aqua and c-PARP.
(3.13 MB TIF)

Table S1 Patient demographics and clinical characteristics. All 25 NRs represent primary refractory AML. The “other” values for race are black and Hispanic subgroups. *Poor prognosis samples have one or more high risk features: age ≥60 years, unfavorable cytogenetics, FLT3-ITD positive or secondary AML. Criteria for sample inclusion in this study: diagnostic prior to initiation of chemotherapy, AML classification by French American British (FAB) criteria as M0 through M7 (and excluding M3) and data for clinical response to induction therapy.
(0.07 MB PDF)

Table S2 Antibodies and reagents used in study.
(0.25 MB PDF)

Table S3 Fluorochrome conjugated antibodies used to measure intracellular pathway activity.
(0.05 MB PDF)

Table S4 Jak/Stat and PI3K pathway nodes that stratified patient response to chemotherapy (NRs versus CRs). Criteria for nodes to stratify patient responses: p-Value <0.1, AUC >0.6. Filters for induced signaling: Fold metric  =  Log2 Mean Fold Signaling >0.25, Total metric  =  when the Pearson correlation between basal and induced signal is <0.75.
(0.08 MB PDF)

Table S5 Quantification of pathways in blast 1 and blast 2 populations from samples depicted in Figures 6A and 6B.
(0.08 MB PDF)
