# Supplementary figures and images for: Distinct Patterns of DNA Damage Response and Apoptosis Correlate with Jak/Stat and PI3Kinase Response Profiles in Human Acute Myelogenous Leukemia
Source: PLoS One. 2010 Aug 25;5(8):e12405. doi: 10.1371/journal.pone.0012405 (PMC2928279; doi:10.1371/journal.pone.0012405)

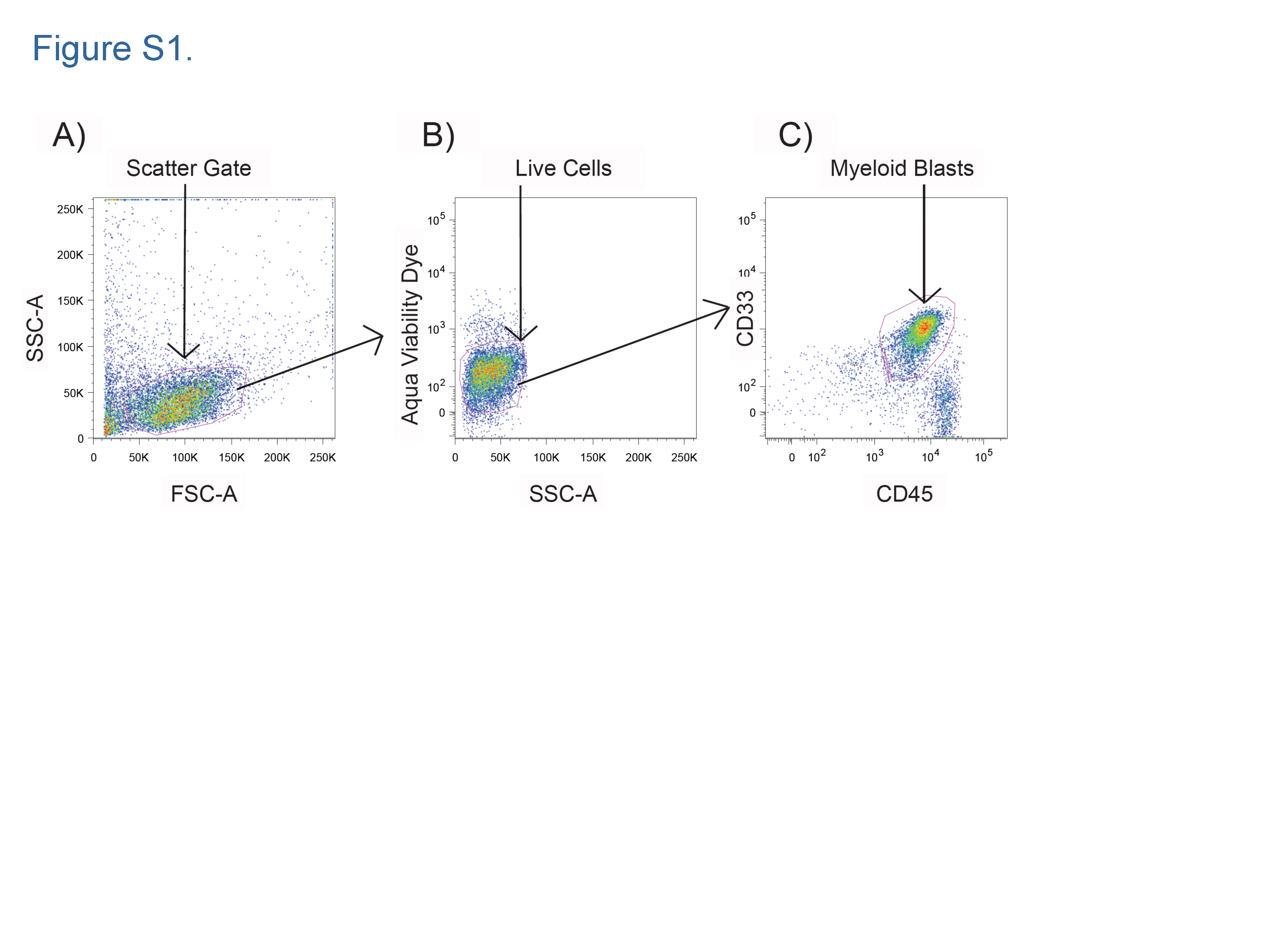

Supplement: Figure S1 — Sequential gating scheme shown by 2D flow plots A) Non-cellular debris was excluded using a FSC and SSC gate. B) Non-viable cells were excluded with a SSC and aqua viability dye gate. C) Leukemic blasts were gated by CD45, CD33. (2.10 MB TIF) [file pone.0012405.s001.tif]

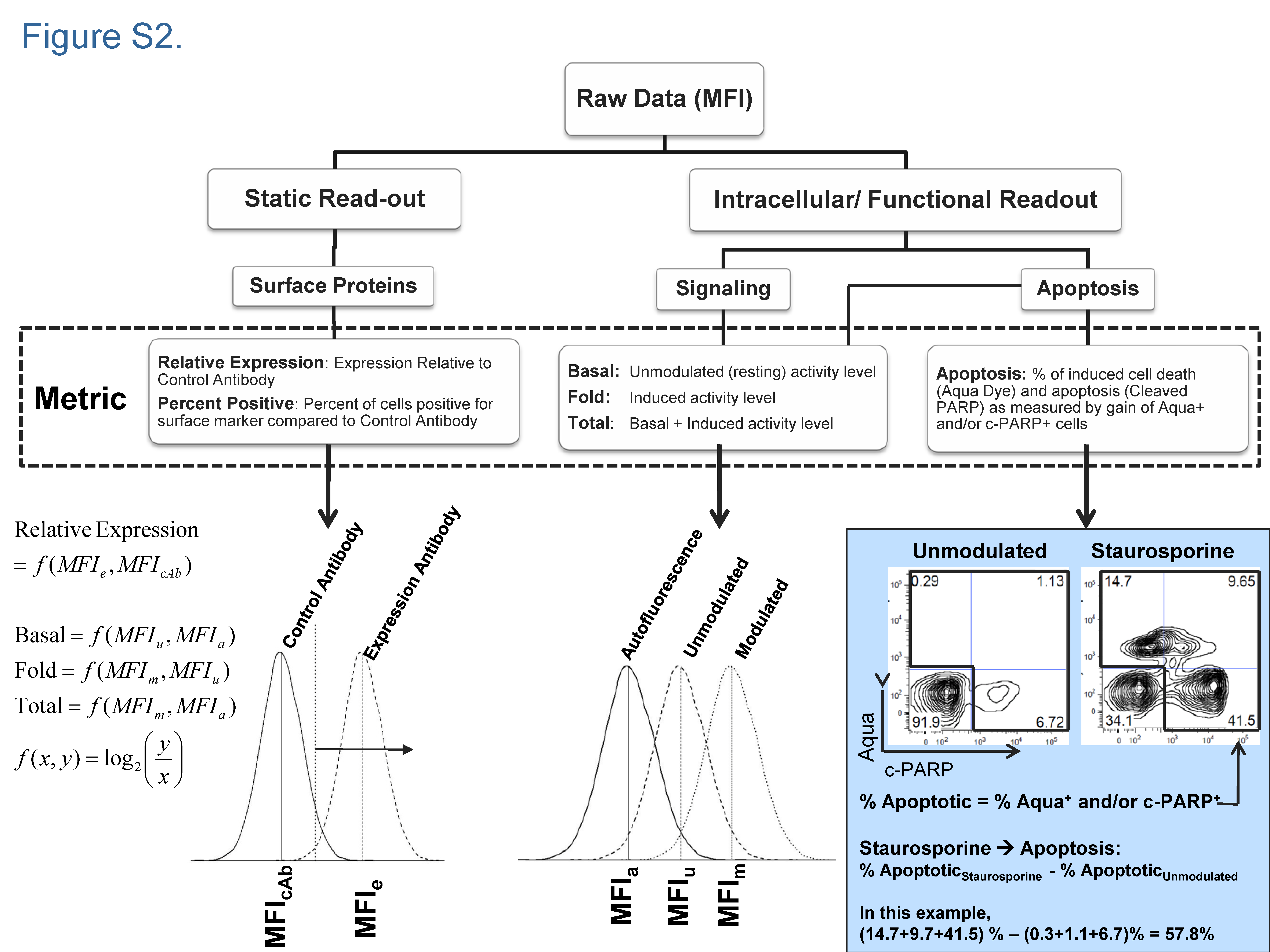

Supplement: Figure S2 — Definition of Metrics A) Summary of metrics used and the role each has in determining a different measure of signaling biology. Median Fluorescence Intensities (MFI) were calculated for leukemic blast cells under each condition and used to compute the metrics that represented protein expression, signaling and apoptosis, as described in Materials and Methods. Apoptosis was measured by levels of c-caspase 3, c-PARP as well as cells that were positive for the aqua viability dye. Measurements for cleaved caspase 3 were found to be correlated with cleaved PARP, but this was not the case for cleaved PARP and aqua. Therefore apoptosis metrics incorporated measurements for aqua and cleaved PARP to quantify cells that became positive for c-PARP alone or for both aqua and c-PARP. (3.13 MB TIF) [file pone.0012405.s002.tif]
