# Supplementary material for: Distinct Patterns of DNA Damage Response and Apoptosis Correlate with Jak/Stat and PI3Kinase Response Profiles in Human Acute Myelogenous Leukemia
Source: PLoS One. 2010 Aug 25;5(8):e12405. doi: 10.1371/journal.pone.0012405 (PMC2928279; doi:10.1371/journal.pone.0012405)
Supplement: Table S1 — Patient demographics and clinical characteristics. All 25 NRs represent primary refractory AML. The “other” values for race are black and Hispanic subgroups. *Poor prognosis samples have one or more high risk features: age ≥60 years, unfavorable cytogenetics, FLT3-ITD positive or secondary AML. Criteria for sample inclusion in this study: diagnostic prior to initiation of chemotherapy, AML classification by French American British (FAB) criteria as M0 through M7 (and excluding M3) and data for clinical response to induction therapy. (0.07 MB PDF) [file pone.0012405.s003.pdf]

Table S1.

Characteristic		CR Patients	NR Patients	All Patients
	N	9	25	34
Age (yr)	Median	57	47.4	49.1
	Range	38.2 - 74.8	20.7 - 70.2	20.7 - 74.8
Age Group	< 60 yr	5 (56%)	20 (80%)	25 (74%)
	>= 60 yr	4 (44%)	5 (20%)	9 (26%)
Sex	F	7 (78%)	14 (56%)	21 (62%)
	M	2 (22%)	11 (44%)	13 (38%)
Cytogentic Group	Favorable	0 (0%)	1 (4%)	1 (3%)
	Intermediate	8 (89%)	18 (72%)	26 (76%)
	Unfavorable	0 (0%)	3 (12%)	3 (9%)
	Not Done	1 (11%)	3 (12%)	4 (12%)
FAB	M0	0 (0%)	2 (8%)	2 (6%)
	M1	2 (22%)	2 (8%)	4 (12%)
	M2	1 (11%)	5 (20%)	6 (18%)
	M4	1 (11%)	7 (28%)	8 (24%)
	M5	3 (33%)	2 (8%)	5 (15%)
	M6	0 (0%)	0 (0%)	0 (0%)
	Other & Unknown	2 (22%)	7 (28%)	9 (27%)
Race	White	3 (33%)	17 (68%)	20 (59%)
	Asian	5 (56%)	5 (20%)	10 (29%)
	Other*	1 (11%)	2 (8%)	3 (9%)
	Unknown	0 (0%)	1 (4%)	1 (3%)
FLT3 -ITD	Negative	4 (44%)	14 (56%)	18 (53%)
	Positive	5 (56%)	10 (40%)	15 (44%)
	Unknown	0 (0%)	1 (4%)	1 (3%)
Secondary AML	No	8 (89%)	25 (100%)	33 (97%)
	Yes	1 (11%)	0 (0%)	1 (3%)
Poor Prognosis†	No	5 (56%)	18 (72%)	23 (68%)
	Yes	4 (44%)	7 (28%)	11 (32%)
Induction Therapy	Standard 3 + 7	9 (100%)	25 (100%)	34 (100%)
