# Supplementary material for: Distinct Patterns of DNA Damage Response and Apoptosis Correlate with Jak/Stat and PI3Kinase Response Profiles in Human Acute Myelogenous Leukemia
Source: PLoS One. 2010 Aug 25;5(8):e12405. doi: 10.1371/journal.pone.0012405 (PMC2928279; doi:10.1371/journal.pone.0012405)
Supplement: Table S2 — Antibodies and reagents used in study. (0.25 MB PDF) [file pone.0012405.s004.pdf]

Table S2

Modulator	Final Concentration	Treatment Duration	Manufacturer
Etoposide	30 µg/ml	24 hours	Sigma Aldrich (St Louis, MO)
FCS	1%	various	HyClone (Waltham, MA)
FLT3L	50 ng/ml	15 mins	eBiosciences (San Diego, CA)
G-CSF*	50 ng/ml	15 mins	R&D (Minneapolis, MN)
G-CSF*	50 ng/ml	15 mins	Pepro (Rocky Hill, NJ)
IFNα	10000 IU/ml	15 mins	Schering (Kenilworth, NJ)
IFNγ	5 ng/ml	15 mins	BD (San Jose, CA)
IL-10	25 ng/ml	15 mins	BD (San Jose, CA)
IL-27	50 ng/ml	15 mins	R&D (Minneapolis, MN)
IL-6	25 ng/ml	15 mins	R&D (Minneapolis, MN)
PMA	400 nM	15 mins	Sigma Aldrich (St Louis, MO)
SCF	20 ng/ml	15 mins	R&D (Minneapolis, MN)
SDF-1α	2 ng/ml	3 mins	R&D (Minneapolis, MN)
Staurosporin	2.33 µg/ml	6 hours	Sigma Aldrich (St Louis, MO)
Thapsigargin	1 µM	15 mins	EMD Biosciences (Darmstadt, Germany)

Antibody	Species & Isotype	Manufacturer	Clone
CD33†	Mouse IgG_1	BD (San Jose, CA)	P67.6
CD45	Mouse IgG_1	Invitrogen (Carlsbad, CA)	HI30
c-Caspase 3	Rabbit IgG	BD (San Jose, CA)	C92-605
c-PARP(Asp214)	Mouse IgG_1, k	BD (San Jose, CA)	F21-852
Goat anti-rabbit secondary	Goat IgG	Invitrogen (Carlsbad, CA)	Polyclonal
p-Akt (S473)	Rabbit IgG	CST (Danvers, MA)	193H12
p-Chk2 (T68)	Rabbit IgG	CST (Danvers, MA)	Polyclonal
p-Erk 1/2 (T202/204)	Mouse IgG_1	BD (San Jose, CA)	20A
p-S6 (S235/236)	Rabbit IgG	CST (Danvers, MA)	2F9
p-Stat1 (pY701)	Mouse IgG_{2a}	BD (San Jose, CA)	4a
p-Stat3 (pY705)	Mouse IgG_{2a}, k	BD (San Jose, CA)	4/p-Stat3
p-Stat5 (pY694)	Mouse IgG_1	BD (San Jose, CA)	47
p-Stat6 (pY641)	Mouse IgG_{2a}	BD (San Jose, CA)	18/p-Stat6
**Non-Antibody Stains**	**n/a**	**Manufacturer**	**n/a**
Amine aqua viability dye	n/a	Invitrogen (Carlsbad, CA)	n/a
Streptavidin-Qdot 605	n/a	Invitrogen (Carlsbad, CA)	n/a

* Products displayed similar bioactivity
