# Supplementary material for: Distinct Patterns of DNA Damage Response and Apoptosis Correlate with Jak/Stat and PI3Kinase Response Profiles in Human Acute Myelogenous Leukemia
Source: PLoS One. 2010 Aug 25;5(8):e12405. doi: 10.1371/journal.pone.0012405 (PMC2928279; doi:10.1371/journal.pone.0012405)
Supplement: Table S3 — Fluorochrome conjugated antibodies used to measure intracellular pathway activity. (0.05 MB PDF) [file pone.0012405.s005.pdf]

Table S3.

Pathway	Modulator	Stain: AF488 or FITC	Stain: PE	Stain: AF4647 or APC
Apoptosis	Etoposide	BCL-2	Cleaved PARP	p-Chk2 (T68)
Apoptosis	Etoposide	Cleaved Caspase 3	Cleaved PARP	None
Apoptosis	Staurosporine	BCL-2	Cleaved PARP	Cleaved Caspase 8
Apoptosis	Staurosporine	Cleaved Caspase 3	Cleaved PARP	None
Jak/Stat	G-CSF	p-Stat1 (Y701)	p-Stat3 (Y705)	p-Stat5 (Y694)
Jak/Stat	IFNα	p-Stat1 (Y701)	p-Stat3 (Y705)	p-Stat5 (Y694)
Jak/Stat	IFNγ	p-Stat1 (Y701)	p-Stat3 (Y705)	p-Stat5 (Y694)
Jak/Stat	IL-10	p-Stat1 (Y701)	p-Stat3 (Y705)	p-Stat5 (Y694)
Jak/Stat	IL-27	p-Stat1 (Y701)	p-Stat3 (Y705)	p-Stat5 (Y694)
Jak/Stat	IL-6	p-Stat1 (Y701)	p-Stat3 (Y705)	p-Stat5 (Y694)
Jak/Stat	None		p-Stat6 (Y641)	p-Stat5 (Y694)
PI3K	FLT3L	p-S6 (S235/236)	p-Erk (T202/Y204)	p-Akt (S473)
PI3K	PMA	p-S6 (S235/236)	p-Creb (S133)	p-Erk (T202/Y204)
PI3K	SCF	p-S6 (S235/236)	p-Erk (T202/Y204)	p-Akt (S473)
PI3K	Thapsigargin	p-S6 (S235/236)	p-Creb (S133)	p-Erk (T202/Y204)
PI3K	SDF-1α	p-S6 (S235/236)	p-Erk (T202/Y204)	p-Akt (S473)
