# Supplementary material for: Distinct Patterns of DNA Damage Response and Apoptosis Correlate with Jak/Stat and PI3Kinase Response Profiles in Human Acute Myelogenous Leukemia
Source: PLoS One. 2010 Aug 25;5(8):e12405. doi: 10.1371/journal.pone.0012405 (PMC2928279; doi:10.1371/journal.pone.0012405)
Supplement: Table S4 — Jak/Stat and PI3K pathway nodes that stratified patient response to chemotherapy (NRs versus CRs). Criteria for nodes to stratify patient responses: p-Value <0.1, AUC >0.6. Filters for induced signaling: Fold metric = Log2 Mean Fold Signaling >0.25, Total metric = when the Pearson correlation between basal and induced signal is <0.75. (0.08 MB PDF) [file pone.0012405.s006.pdf]

Table S4.

Node	Metric	pval (t-test)	pval (Wilcox)	AUC of ROC	Mean Signal CR	Mean Signal NR
**Jak/Stat Pathway Nodes**						
**G-CSF → p-Stat3**	Total	0.056	0.050	0.72	1.66	2.70
**G-CSF → p-Stat3**	Fold	0.091	0.111	0.68	0.81	1.48
**G-CSF → p-Stat5**	Fold	0.038	0.072	0.71	0.47	1.13
**IFNα → p-Stat1**	Fold	0.017	0.030	0.75	0.55	0.78
**IFNγ → p-Stat1**	Total	0.054	0.055	0.72	0.76	1.26
**IFNγ → p-Stat1**	Fold	0.039	0.072	0.71	0.53	0.90
**IL-10 → p-Stat3**	Total	0.001	0.002	0.84	0.82	1.69
**IL-27 → p-Stat1**	Total	0.001	0.003	0.83	0.41	0.82
**IL-27 → p-Stat3**	Total	0.000	0.000	0.90	1.07	1.86
**IL-6 → p-Stat3**	Total	0.001	0.015	0.77	1.08	1.84
**IL-6 → p-Stat3**	Fold	0.082	0.329	0.61	0.17	0.50
**Basal p-Stat3**	Total	0.005	0.005	0.81	0.89	1.33
**Basal p-Stat6**	Total	0.008	0.019	0.76	0.62	0.96
**PI3K Pathway Nodes**						
**FLT3L → p-Akt**	Fold	0.003	0.004	0.82	0.18	0.64
**FLT3L → p-Erk**	Fold	0.056	0.053	0.72	0.13	0.32
**FLT3L → p-S6**	Total	0.057	0.397	0.60	0.60	1.15
**FLT3L → p-S6**	Fold	0.026	0.154	0.66	0.28	0.81
**SCF → p-Akt**	Total	0.053	0.202	0.65	0.62	1.09
**SCF → p-Akt**	Fold	0.018	0.007	0.81	0.12	0.57
**SCF → p-S6**	Total	0.038	0.355	0.61	0.43	0.85
**SCF → p-S6**	Fold	0.055	0.163	0.66	0.11	0.51
**PMA → p-Erk**	Fold	0.063	0.079	0.70	1.54	2.02
**PMA → p-S6**	Total	0.083	0.151	0.67	1.46	1.98
**SDF-1α → p-Akt**	Total	0.045	0.120	0.68	0.57	1.04
**SDF-1α → p-Akt**	Fold	0.025	0.067	0.71	0.20	0.53
**Thapsigargin → p-S6**	Total	0.018	0.045	0.73	0.31	0.68
**Basal→ p-Erk**	Total	0.028	0.015	0.77	1.69	2.09
