# Supplementary material for: Distinct Patterns of DNA Damage Response and Apoptosis Correlate with Jak/Stat and PI3Kinase Response Profiles in Human Acute Myelogenous Leukemia
Source: PLoS One. 2010 Aug 25;5(8):e12405. doi: 10.1371/journal.pone.0012405 (PMC2928279; doi:10.1371/journal.pone.0012405)
Supplement: Table S5 — Quantification of pathways in blast 1 and blast 2 populations from samples depicted in Figures 6A and 6B. (0.08 MB PDF) [file pone.0012405.s007.pdf]

Table S5.

Donor	FLT3-ITD	Response	Population	FAB	sex	race	Cyto	Stauro → c-PARP | %	Stauro → cCasp-3 | %	Stauro → Apoptosis	Etopo → cPARP | %	Etopo → cCasp-3 | %	Etoposide → Apoptosis	Etopo → p-Chk2 | Fold*	Etopo → p-Chk2 | %*	Etoposide → DNA Damage
UHN_0577	WT	NR	Blast 1	n.d.	f	w	INT-risk	58.8	77.4	+	1.1	13.8	-	2	54.1	++
			Blast 2	n.d.	f	w	INT-risk	68.7	65.1	+	8.9	12.7	-	1.75	32.7	+
UHN_8093	ITD	NR	Blast 1	m4	f	ind	INT-risk	44.7	73.7	+	0.8	5	-	1.66	34.3	+
			Blast 2	m4	f	ind	INT-risk	17	13.8	-	13.2	5.8	-	1.34	2.8	-

Donor	FLT3-ITD	Response	Population	Unstim → p-S6 | Basal	FLT3L → p-S6 | Total	FLT3L → p-S6 | Fold	Unstim → p-Akt | Basal	FLT3L → p-Akt | Total	FLT3L → p-Akt | Fold	Unstim → p-Stat3 | Basal	G-CSF → p-Stat3 | Total	G-CSF → p-Stat3 | Fold	Unstim → p-Stat5 | Basal	G-CSF → p-Stat5 | Total	G-CSF → p-Stat5 | Fold
UHN_0577	WT	NR	Blast 1	1.94	3.34	1.03	2.44	3.39	0.75	1.43	0.98	-0.16	2.54	1.64	-0.13
			Blast 2	0.3	2.79	2.46	1.25	1.74	0.34	1.71	1.45	-0.06	2.12	1.24	-0.15
UHN_8093	ITD	NR	Blast 1	0.43	0.63	0.33	0.46	1.29	0.92	1.41	2.36	1.85	1.72	1.26	1.67
			Blast 2	0.15	0.83	0.71	0.16	0.99	0.78	0.71	2.65	2.32	0.92	2.22	2.33

* Assessed in c-PARP-
